# Potential Wound Healing and Anti-Melanogenic Activities in Skin Cells of *Aralia elata* (Miq.) Seem. Flower Essential Oil and Its Chemical Composition

**DOI:** 10.3390/pharmaceutics16081008

**Published:** 2024-07-30

**Authors:** Do Yoon Kim, Kyung Jong Won, Yoon Yi Kim, Da Yeon Yoo, Hwan Myung Lee

**Affiliations:** 1 Department of Biotechnology, College of Life and Health Sciences, Hoseo University, Asan 31499, Republic of Korea; doyoon@hoseo.edu (D.Y.K.); yoontwo22@gmail.com (Y.Y.K.); dbekdus111@naver.com (D.Y.Y.); 2 Korea Essential Oil Resource Research Institute, Hoseo University, Asan 31499, Republic of Korea; 3 Department of Physiology and Premedical Science, College of Medicine, Konkuk University, Chungju 27478, Republic of Korea; kjwon@kku.ac.kr

**Keywords:** *Aralia elata* (Miq.) Seem., essential oil, skin wound healing, skin whitening, anti-melanogenesis, keratinocytes, melanocytes

## Abstract

*Aralia elata* (Miq.) Seem. (AES; family Araliaceae) is a medicinal plant and has been reported to have various bioactivities, including anticancer and hepatotoxicity protective activities. However, no studies have investigated the biological activities of AES or its extracts on skin. To address this, we aimed to explore the effect of AES-flower-derived absolute-type essential oil (AESFEO) on skin-related biological activities, especially skin wound healing and whitening-related responses in skin cells (human-derived keratinocytes [HaCaT cells] and melanocytes [B16BL6 cells]) and to identify the components of AESFEO. Cell biological activities were analyzed using WST and BrdU incorporation assays, ELISA, or by immunoblotting. In HaCaT cells, AESFEO promoted proliferation, type IV collagen production, and enhanced the phosphorylations of Erk1/2, p38 MAPK, JNK, and Akt. In B16BL6 cells, AESFEO reduced serum-induced proliferation, α-MSH-stimulated increases in melanin synthesis and tyrosinase activity, and α-MSH-induced increases in MITF, tyrosinase, TRP-1, and TRP-2 expressions. In addition, AESFEO inhibited the phosphorylation of Erk1/2, p38 MAPK, and JNK in α-MSH-stimulated B16BL6 cells. Eighteen compounds were identified in AESFEO by GC/MS. These results suggest that AESFEO has beneficial effects on keratinocyte activities related to skin wound healing and melanocyte activities related to inhibition of skin pigmentation. AESFEO may serve as a useful natural substance for developing agents that facilitate skin wound healing and inhibit melanogenesis.

## 1. Introduction

Human skin acts as the first defense line against external threats by providing a protective barrier that protects the body from pathogens and harmful agents [1]. Therefore, the rapid and effective healing of skin wounds is a complex and dynamic process essential for restoring skin barrier integrity and function [1,2]. The healing process of skin wounds involves a cascade of events orchestrated by various cellular and molecular components [2]. When skin is damaged, keratinocytes proliferate and migrate to cover the wounded area during the re-epithelialization stage and play a central role in promoting tissue repair and protective barrier formation and function [3]. These actions of keratinocytes during skin wound healing are regulated by intricate signaling pathways, notably the MAPK (mitogen-activated protein kinase) signaling pathway, which includes Erk1/2 (extracellular signal-regulated kinase 1/2), p38 MAPK, and JNK (c-Jun N-terminal kinase), and/or the Akt (serine/threonine-specific protein kinase) signaling pathway [4,5,6,7]. Furthermore, collagen, the primary structural component of ECM, has pleiotropic functions during skin wound healing and plays a critical role in the proliferation and remodeling phase [8,9]. Of twenty-eight known collagen types, collagen types I and IV are known to contribute to skin recovery and skin membrane formation, respectively. They were also shown to be involved in the migration and proliferation activities of keratinocytes [9,10,11]. This suggests that modulation of these keratinocyte activities could be a promising approach for promote the healing of skin wounds.

Melanin is responsible for skin color and provides a natural barrier that protects the skin against ultraviolet radiation [12]. However, the dysregulation of melanin synthesis has been implicated in the etiology of abnormal pigmentation, which can lead to various abnormal skin conditions, including melasma, age spots, solar lentigo, and freckles [13]. Melanin synthesis, that is, melanogenesis, occurs within melanosomes in melanocytes and is mediated by complex enzymatic and biochemical reactions by various external stimuli [12]. The synthesis of melanin commences with the oxidation of L-tyrosine to L-3,4-dihydroxyphenylalanine (L-DOPA) by tyrosinase, making the pivotal rate-limiting step in melanogenesis. L-DOPA is subsequently oxidized by tyrosinase to dopaquinone, which is converted to dopachrome by redox exchange between it and cyclodopa and L-DOPA [13,14]. TRP-2 (tyrosinase-related protein-2) then converts dopachrome into DHICA (5,6-dihydroxyindole-2-carboxylic acid), and TRP-1 catalyzes the conversion of DHICA to indole-5,6-quinone-2-carboxylic acid to yield melanin [14]. Thus, tyrosinase, TRP-1 and TRP-2 are key regulators of melanogenesis [13]. MITF (microphthalmia-associated transcription factor) is a major regulator of tyrosinase, TRP-1, and TRP-2, and thus, has a major influence on melanin synthesis [12]. Moreover, MITF is activated by signaling pathways involving MAPKs that stimulate melanogenesis in response to various external stimuli in melanocytes [12,13]. Therefore, regulating these players in melanocytes might provide therapeutic strategies that target skin pigmentation disorders or provide a means of whitening skin.

Various agents have been developed by the cosmetic and dermatological industries to facilitate skin wound healing and/or skin whitening [15,16]. However, some of these synthetic agents have been reported to cause adverse effects, such as skin irritation and allergic reactions [15,17]. Numerous plants and plant extracts provide a rich source of bioactive compounds with various therapeutic benefits but fewer side effects than synthetic chemicals [18]. Thus, there is growing interest in using plants or plant extracts to develop agents for skin whitening and wound healing.

*Aralia elata* (Miq.) Seem. (AES) is a member of the Araliaceae family and has long been used as a medicinal and edible plant in Japan, Korea, and China [19]. The young shoots of AES are popular wild vegetables due to their nutritional value and delicious taste [19,20]. AES, especially its roots, bark, and leaves, has traditionally been utilized as a medicinal herb to treat a variety of disorders and health conditions [19]. AES has been reported to have beneficial biological activities, which include antitumor, anti-myocardial ischemia, hypoglycemic, lipid lowing, neuroprotective, anti-inflammatory, and hepatotoxicity protective effects [20,21,22,23]. However, the effects of AES and its extracts on wound healing and anti-melanogenesis have not been investigated. Thus, in the present study, we extracted AES flower absolute-type essential oil (AESFEO) and investigated its influences on skin wound healing and whitening-linked activities using HaCaT human keratinocytes and B16BL6 murine melanoma cells, respectively. Furthermore, we performed chemical composition analysis of AESFEO by GC/MS (gas chromatography/mass spectrometry).

## 2. Materials and Methods

### 2.1. Materials

Minimum essential medium (MEM), phosphate-buffered saline (PBS) and Dulbecco’s modified eagle medium (DMEM) were purchased from Welgene (Daegu, Republic of Korea). Penicillin/streptomycin (P/S), fetal bovine serum (FBS). Trypsin-ethylenediamine tetra-acetic acid (EDTA) were obtained from Gibco BRL (Gaithersburg, MD, USA). Triton X-100, α-melanocyte stimulating hormone (α-MSH), L-DOPA, bovine serum albumin (BSA), dimethyl sulfoxide (DMSO), and phenylmethylsulfonyl fluoride were supplied by MilliporeSigma (St. Louis, MO, USA). EZ-CyTox kits were obtained from DoGenBio (Seoul, Republic of Korea). Recombinant human epidermal growth factor (EGF: purity > 97%) was obtained from R&D Systems (Minneapolis, MN, USA). The antibodies used were purchased from various companies: monoclonal anti-type IV collagen and monoclonal anti-type I collagen from Invitrogen (Carlsbad, CA, USA); polyclonal anti-type I, polyclonal anti-IV collagen, anti-tyrosinase, anti-TRP-1, and anti-TRP-2 from Abcam (Cambridge, UK); anti-MITF, anti-mouse IgG, anti-rabbit IgG, anti-Akt, anti-JNK, anti-p38 MAPK, anti-Erk1/2, anti-phospho Akt, anti-phospho JNK, anti-phospho p38 MAPK, and anti-phospho Erk1/2 from Cell Signaling (Beverly, MA, USA); and β-actin from MilliporeSigma.

### 2.2. Extraction of Aralia elata (Miq.) Seem. Flower Absolute-Type Essential Oil

AES flowers were collected from plants grown in Hanaro Farm (latitude 34°23′00.4″ N, longituce 126°33′59.0″ E; located in Songji-myeon, Jeollanam-do, Republic of Korea) on 12 August 2017. Their identification was verified by Jong-Cheol Yang at the Baekdudaegan National Arboretum, Republic of Korea. A voucher specimen (No. AES-001) was kept at the Herbarium of the Korea Essential Oil Resource Research Institute, which is located at Hoseo University in Republic of Korea. Extraction of absolute-type essential oil was performed using a previously reported solvent extraction method [24]. In brief, AES flowers (5.345 kg) were placed in hexane (Samchun Chemicals, Pyeongtaek, Republic of Korea) and left at room temperature (RT) for 1 h. The crude hexane extracts were collected, and the hexane was subsequently evaporated at 25 °C under vacuum, resulting in a dark-yellow waxy residue (concrete). This residue was dissolved in ethanol (purity 99.5%; Samchun Chemicals, Pyungtaek, Republic of Korea and kept at −20 °C for 12 h. After filtration through a sintered funnel, the ethanol was evaporated at 35 °C, producing a light-yellow anhydrous wax (AESFEO; 2.8 g, yield 0.052%, *w*/*w*). The AESFEO obtained was stored at −80 °C until required.

### 2.3. Compounds Analysis in Aralia elata (Miq.) Seem. Flower Absolute-Type Essential Oil

The chemical composition of AESFEO was analyzed at the Korean Basic Science Institute (KBSI) in Seoul, Republic of Korea, as in a previous study [24]. Briefly, GC/MS analysis was conducted using an Agilent 7890BGC/7010QQQ MS system (Palo Alto, CA, USA) fitted with a DB5-MS capillary column (30 m in length and 250 μm in internal diameter) with a film thickness of 0.25 μm. Helium was employed as the carrier gas and its flow rate was 1 mL/min. The injector port and interface were both maintained at a temperature of 300 °C. The GC oven was programmed as follows: 40 °C for 2 min, 40 to 230 °C at 2 °C/min, 230 to 300 °C at 5 °C/min, and 300 °C for 15 min. The GC/MS analysis utilized a split ratio of 1:10, with masses scanning in a range of *m*/*z* 50 to 800. Retention indices (RIs) were calculated using Kovats method with standard C_7_-C_40_ n-alkanes. Compound identification was achieved by comparing the obtained RI values with Kovats indices [25] and by matching the mass spectra fragmentation patterns against those in the Wiley7NIST0.5L Mass Spectral library and additional mass spectra catalogs. The re-analysis of GC/MS followed the methodology of Adams [26].

### 2.4. Cell Culture

HaCaT cells, which are a human keratinocyte cell line, were sourced from the National Institute of Korean Medicine Development (NIKOM) in Gyeongsan, Republic of Korea. Additionally, B16BL6 cells, which are a murine melanoma cell line, were provided by the Intercellular Communication Network Lab at POSTECH in Pohang, Republic of Korea. HaCaT cells were cultured in DMEM with 10% FBS and 1% P/S, while B16BL6 cells were cultured in MEM with the same supplements. Both cell types were cultured for use in experiments until 70–80% confluence under humidified conditions at 37 °C with 5% air and 5% CO_2_.

### 2.5. Cell Viability Assay

Cell viability was analyzed using a WST (water-soluble tetrazolium salt) assay using EZ-CyTox kits (DoGenBio). The cells (HaCaT cells, 3 × 10^3^ cells/well; B16BL6 cells, 2 × 10^3^ cells/well) were plated into 96-well microtiter plates and subsequently exposed to varying concentrations of AESFEO for 48 h. AESFEO was dissolved in DMEM with 0.5% DMSO for HaCaT cells. For B16BL6 cells, it was dissolved in MEM with 2% FBS along with 0.5% DMSO. The cells were then treated with EZ-CyTox reagent (10 μL/well) and maintained for 30 min at 37 °C. Measurements of absorbance were conducted at 450 nm using the Synergy 2 multi-well plate reader (Bio-Tek Instruments, Winooski, VT, USA).

### 2.6. Proliferation Assay

HaCaT cell proliferation was evaluated by analyzing a BrdU (5-bromo-2′-deoxyuridine) incorporation with a BrdU kit obtained from Roche (Indianapolis, IN, USA) and ELISA [27]. In brief, the cells were seeded in 96-well plates at a density of 3 × 10^3^ cells per well and incubated for 12 h. The cells were treated with AESFEO at varying concentrations of or 50 ng/mL EGF for 36 h. Subsequently, BrdU-labeling solution (10 μM) was added, and the cells were further incubated for 12 h at 37 °C. After discarding the culture medium, the cells were fixed and subjected to the DNA denaturation by exposure to FixDenat solution included in the BrdU kit for 30 min. Subsequently, peroxidase labeled anti-BrdU monoclonal antibody was introduced and allowed to incubate for 90 min at RT. The luminescent signal from the reaction was assessed with a luminometer (Synergy 2; Bio-Tek Instruments).

B16BL6 cell proliferation was determined by a WST assay using EZ-CyTox kits (DoGenBio). The cells were seeded in 96-well microtiter plates at 2 × 10^3^ cells/well and then treated with different concentrations of AESFEO for 48 h. AESFEO was dissolved in MEM with 0.5% DMSO and 2% FBS. The cells were then exposed to EZ-CyTox reagent (10 μL/well) for 30 min at 37 °C. Absorbance readings were then obtained at 450 nm utilizing a Synergy 2 multi-well plate reader (Bio-Tek Instruments).

### 2.7. Cell Migration Assay

HaCaT cell migration was assessed utilizing a 48-well microchemotaxis Boyden chamber from Neuro Probe Inc. (Gaithersburg, MD, USA), following previous protocols [27]. In brief, in the lower chamber wells, DMEM supplemented with 0.1% BSA and 1 ng/mL EGF or various concentrations of AESFEO was added for loading. A membrane, precoated type Ι collagen (BD Bioscience, Franklin Lakes, NJ, USA) at 0.1 mg/mL, was then positioned over the lower chamber wells. HaCaT cells in DMEM supplemented with 0.1% BSA were then loaded into the upper chambers at a density of 5 × 10^4^ cells/well. After assembly, the chambers were maintained for 210 min at 37 °C, and then the membranes were taken out, fixed, and stained using the Diff-Quick staining protocol provided by the manufacturer (Baxter Healthcare Miami, FL, USA). The quantity of cells that migrated onto the lower membrane surface was determined using an optical microscope (Carl Zeiss, Jena, Thuringen, Germany) (×200).

### 2.8. Collagen Synthesis Assay

Collagen synthesis was assessed using ELISA, following the procedures of a previous study [24]. Briefly, HaCaT cells were plated in 6-well plates at 2 × 10^5^ cells/well and incubated in media with various concentrations of AESFEO for 48 h at 37 °C. The culture media were subjected to sequential centrifugation at 500, 800, and 1000× *g* for 10 min each. The supernatants obtained (termed conditioned media) were then added at 100 μL/well to 96-well microtiter plates coated with specific capture antibodies (monoclonal antibodies against type I or IV collagen). Subsequently, they were incubated with biotin-conjugated polyclonal antibody specific to collagen type I or IV (diluted 1:2000 in 1% BSA/PBS) for 90 min at RT. After PBS wash, the wells were filled with streptavidin–horseradish peroxidase conjugate (Roche) at a 1:5000 dilution in 1% BSA/PBS, and left to incubate for 1 h at RT. Subsequently, the wells were washed again with PBS. An enhanced chemiluminescence (ECL) solution from Thermo Fisher Scientific (Waltham, MA, USA) was introduced, followed by measuring the luminescence emitted by reaction products with a Synergy 2 luminometer (Bio-Tek Instruments).

### 2.9. Western Blotting

The cells were lysed using RIPA buffer (Cell Signaling), and then subjected to centrifugation at 17,000× *g* for 15 min at 4 °C to obtain supernatants. The concentrations of proteins in the supernatants were assessed using the DC protein assay kits from Bio-Rad Laboratories (Hercules, CA, USA). In addition, the proteins (60–120 μg per lane) underwent separation using electrophoresis on a 10% polyacrylamide gel containing sodium dodecyl sulfate (SDS-PAGE) and transferred to PVDF (polyvinylidene fluoride) membranes at 4 °C. The membranes were then treated to a blocking solution consisting of 3% skim milk at RT for 2 h. Afterward, they underwent washing with PBS with 0.05% Tween-20 and incubated with primary antibodies at a dilution range of 1:1000 to 1:5000. Subsequently, the membranes were exposed to a secondary antibody (conjugated with horseradish peroxidase) at RT for 1 h. Immunoreactive bands were detected using a chemiluminescence-based substrate and captured via an imaging system (LuminoGraph; ATTO, Tokyo, Japan).

### 2.10. Melanin Content Assay

Melanin content levels were assessed using the methods described in a previous report [28]. Briefly, B16BL6 cells at a density of 5 × 10^4^ cells per dish were plated in 6-well plates and cultured for 12 h. Subsequently, the cells were subjected to incubation in MEM (supplemented with 2% FBS) for 48 h at 37 °C. During this incubation, various concentrations of AESFEO were added, with and without of 200 nM α-MSH. The cells were washed with PBS and underwent lysis using a lysis buffer composed of 0.1 M sodium phosphate buffer (pH 6.8), supplemented with Triton X-100 (1%) and phenylmethylsulfonyl fluoride (0.2 mM). After centrifugation at 10,000× *g* for 15 min, cell pellets were collected. The isolated cell pellets were resuspended in 150 μL of a solution consisting of 1 N NaOH with 10% DMSO, followed by an incubation at 80 °C for 1 h. The samples were then pipetted to dissolve melanin, and absorbance readings at 405 nm were recorded with an ELISA reader (Synergy 2; Bio-Tek Instruments).

### 2.11. Tyrosinase Activity Analysis

Tyrosinase activity was analyzed using the dopa chrome method with L-DOPA as the substrate, following previously described procedures [29]. Briefly, B16BL6 cells were cultured at 5 × 10^4^ cells per well in 6-well plates, followed by lysis and centrifugation, as described in the earlier section on melanin content assay. Subsequently, supernatants (60 μL) and 2 mg/mL L-DOPA (140 μL) were dispensed into each of the wells in a 96-well plate and allowed to incubate at 37 °C for 60 min. The absorbance values of the reaction product were determined at 490 nm utilizing the Synergy 2 ELISA reader (Bio-Tek Instruments).

### 2.12. Analysis

Data are expressed as the means ± standard errors of means (SEMs). The significance of differences between pairs of groups was evaluated using Student’s *t*-test, while differences among multiple groups were analyzed through one-way analysis of variance (ANOVA), with subsequent Tukey’s post hoc testing. The analyses were carried out using GraphPad Prism version 5.0 (GraphPad Software Inc., La Jolla, CA, USA). Statistical significance was determined for *p* values lower than 0.05.

## 3. Results and Discussion

### 3.1. Proliferation and Migration of AESFEO-Treated HaCaT Cells

**Figure 1:**
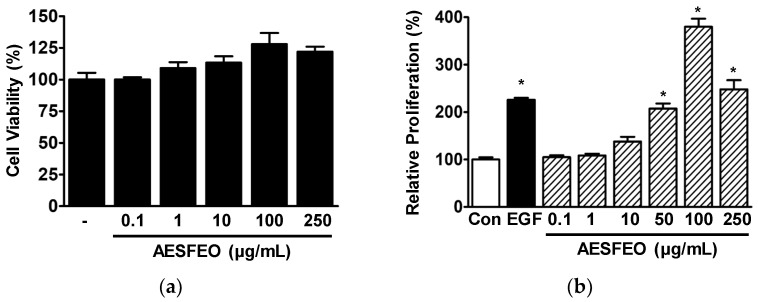
Effects of AESFEO on the proliferation of HaCaT cells. (**a**) Cell viability. Cells were treated with and without *Aralia elata* (Miq.) Seem. flower absolute-type essential oil (AESFEO) at concentrations ranging from 0.1 to 250 µg/mL for 48 h. Cell viability was assessed using the WST assay (n = 3). (**b**) Cell proliferation. Cells were exposed to AESFEO at concentrations ranging from 0.1 to 250 µg/mL for 48 h. Proliferation was measured through the BrdU incorporation assay (n = 3). EGF (Recombinant human epidermal growth factor; 50 ng/mL) indicates the positive control. Proliferations were calculated as percentages of untreated cells (Con). Data represented as means ± SEMs. * *p* < 0.05 vs. untreated cells.

We analyzed the effects of AESFEO on the proliferation and migration responses of keratinocytes to determine whether it might influence skin wound healing. Initially, we used WST assays to investigate the effect of AESFEO on HaCaT cell viability at concentrations from 0.1 to 250 μg/mL. The results showed that AESFEO did not affect HaCaT cell viability at 0.1 μg/mL and tended to increase HaCaT cell viability at concentrations between 1 and 250 μg/mL (Figure 1a), indicating that it has no effect on HaCaT cytotoxicity. Thus, we selected AESFEO test concentrations from 0.1 to 250 μg/mL for other HaCaT cell experiments. The effect of AESFEO on HaCaT cell proliferation was assessed using BrdU incorporation assays. AESFEO significantly elevated HaCaT cell proliferation at concentrations ranging from 50 to 250 μg/mL, and the effect peaked at 100 μg/mL (at 379.66 ± 17.11% of the untreated control level) (Figure 1b). In addition, the effect of AESFEO on HaCaT cell migration was investigated using a Boyden chamber assay. AESFEO (0.1–250 μg/mL) did not influence HaCaT cell migration.

Keratinocytes are the most numerous cells in the epidermis [30], and when skin is injured, they migrate and proliferate to contribute to re-epithelization, which is a crucial process in skin wound healing [31]. In addition, when the skin is damaged, keratinocytes migrate and proliferate, facilitating the re-exfoliation of the epidermis, which is a key process in the healing of skin wounds [32,33,34,35]. Such reports indicate that promoting keratinocyte proliferation and migration might beneficially affect skin wound healing. In the present study, AESFEO promoted the proliferative activity but not the migratory activity of HaCaT cells, suggesting it might promote wound healing by increasing the proliferative activity of keratinocytes.

### 3.2. Influence of AESFEO on Collagen Synthesis in HaCaT Cells

**Figure 2:**
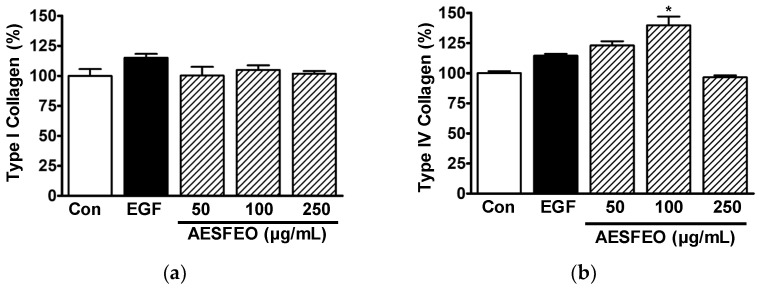
Effects of AESFEO on type I and type IV collagen synthesis in HaCaT cells. Cells were cultured in the presence or absence of *Aralia elata* (Miq.) Seem. flower absolute-type essential oil (AESFEO; 50–250 µg/mL) for 48 h. Collected conditioned media were loaded with antibodies targeting type I (**a**) or type IV collagen (**b**), and sandwich ELISA was performed (n = 3/collagen type). Collagen levels were calculated as percentages of untreated cells (Con). EGF (Recombinant human epidermal growth factor; 50 ng/mL) indicates the positive control. Data are shown as means ± SEMs. * *p* < 0.05 vs. untreated cells.

Collagen type I and IV levels in the conditioned media of HaCaT cells exposed to AESFEO were determined by ELISA. Treatment of HaCaT cells with AESFEO at concentrations of 50 to 250 μg/mL had no effect on the level of collagen type I (Figure 2a), but at 100 μg/mL, it increased the level of collagen type IV significantly, to 139.65 ± 7.35%, compared to that of the untreated control level (Figure 2b).

Type I and IV collagens are known to participate in skin recovery [10] and skin basement membrane formation [9,11], respectively. Furthermore, collagen types I and IV have been reported to be synthesized by keratinocytes exposed to various plant extracts [27,36,37]. In this study, we found that exposure of keratinocytes to AESFEO increased collagen type IV levels but not type I levels, suggesting it might improve wound healing.

### 3.3. AESFEO Induced Changes in Intracellular Signal Kinases in HaCaT Cells

**Figure 3:**
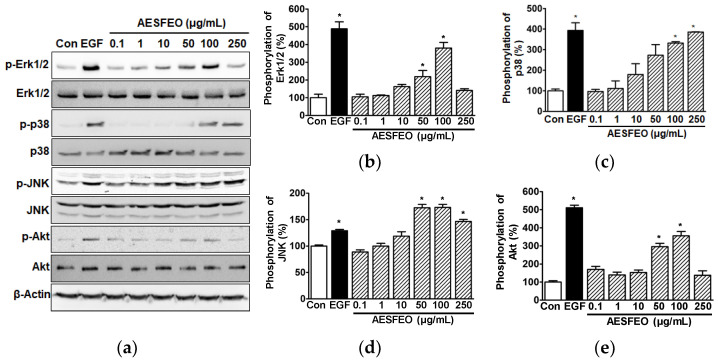
Effect of AESFEO on MAPKs and Akt in HaCaT cells. (**a**) Images of representative immunoblotting results. Cells were subjected to treatment with and without *Aralia elata* (Miq.) Seem. flower absolute-type essential oil (AESFEO; 0.1–250 µg/mL) for 10 min. Cell lysates were Western blotted with antibodies targeting indicated proteins. (**b**–**e**) Graphs of the levels of phosphorylated Erk1/2 (**b**, p-Erk1/2), p38 MAPK (**c**, p-p38), JNK (**d**, p-JNK), and Akt (**e**, p-Akt) shown in panel (**a**). Phosphorylation levels are presented as percentages of levels in untreated cells (Con). The positive control utilized recombinant human epidermal growth factor (EGF) at a concentration of 5 ng/mL. Data are shown as means ± SEMs, with three replicates performed for each protein. * *p* < 0.05 vs. untreated cells.

Studies have indicated that MAPKs and Akt are key signal kinases that mediate the proliferation and migration of HaCaT cells [6,7,38]. We thus analyzed AESFEO-induced phosphorylation alterations in MAPKs and Akt in HaCaT cells by Western blot. AESFEO (0.1–250 μg/mL) significantly elevated phosphorylated Erk1/2 levels at 50 and 100 μg/mL (Figure 3a,b), p38 MAPK at 100 and 250 μg/mL (Figure 3a,c), and JNK at 50–250 μg/mL (Figure 3a,d). The phosphorylation levels of Erk1/2 and JNK peaked after treatment with 100 μg/mL of AESFEO (379.71 ± 32.03% vs. the untreated control [Figure 3b] and 173.21 ± 5.69% vs. the untreated control [Figure 3d], respectively). Furthermore, the phosphorylation level of p38 MAPK peaked after treatment with 250 μg/mL of AESFEO (385.74 ± 0.73% vs. untreated control, Figure 3c). In addition, AESFEO at 50 and 100 μg/mL significantly enhanced Akt phosphorylation, and this peaked at 100 μg/mL (355.29 ± 24.35% vs. the untreated control, Figure 3a,e).

MAPKs regulate diverse cellular responses, including survival, death, migration, proliferation, and differentiation [39]. Erk1/2, p38 MAPK, and JNK are known as the three main subfamilies of MAPKs [39]. Elevated Erk1/2 phosphorylation levels have been demonstrated to contribute to the induction of keratinocyte migration and proliferation [38,40]. Conversely, diminished Erk1/2 phosphorylation levels have been found to reduce keratinocyte proliferation and migration [4,41]. Phosphorylated p38 MAPK functions as a pivotal signaling kinase that activates keratinocyte proliferation and migration [5,7]. Phosphorylated JNK contributes to stimulating the induction of keratinocyte migration and proliferation [6]. Additionally, Akt is a signaling molecule that promotes cellular responses, including proliferation and migration [24,42], and Akt phosphorylation reportedly mediates keratinocyte migratory and proliferative responses [38,40,43]. These reports suggest that MAPKs and Akt function as signaling molecules that mediate keratinocyte migration and proliferative responses. In the present study, we found that AESFEO upregulated the phosphorylation levels of Erk1/2, p38 MAPK, JNK, and Akt in HaCaT cells, though these responses were induced at different concentrations. In addition, AESFEO induced HaCaT cells’ proliferation but not migration. Therefore, our results suggest that AESFEO may induce HaCaT cells’ proliferation by activating MAPKs (p38 MAPK, Erk1/2, and JNK) and/or Akt.

### 3.4. Cell Proliferation, Melanin Synthesis, and Tyrosinase Activity in AESFEO-Treated B16BL6 Cells

**Figure 4:**
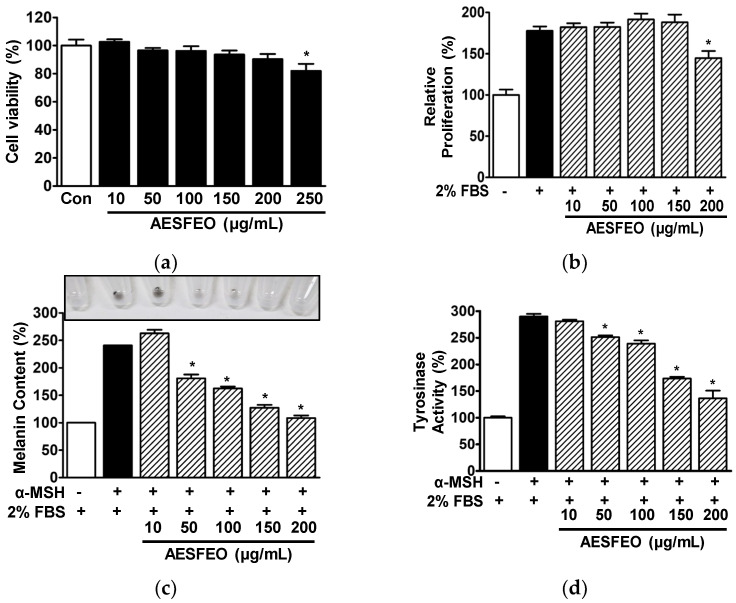
Influences of AESFEO on B16BL6 melanoma cell responses related to skin whitening. (**a**) Viability analysis. B16BL6 cells were treated with and without *Aralia elata* (Miq.) Seem. flower absolute-type essential oil (AESFEO; 10–250 μg/mL) for 48 h. Cell viability was evaluated using a WST assay (n = 3). Results represent percentages of levels in untreated cells (Con). * *p* < 0.05 vs. untreated cells. (**b**) Proliferation analysis. B16BL6 cells were cultured for 48 h with and without AESFEO at varying concentrations (10–200 μg/mL) in the presence or absence of 2% FBS for 48 h. Cell proliferation were assessed through the WST assay method as outlined in the Materials and Methods section (n = 3). Results are presented as percentages of levels in untreated controls. * *p* < 0.05 vs. 2% FBS alone-treated cells. (**c**,**d**) Melanin content and tyrosinase activity analysis. B16BL6 cells were cultured for 48 h in media containing 2% FBS with and without 200 nM α-MSH, along with AESFEO (10–200 μg/mL) or without it. Melanin contents (**c**) and tyrosinase activities (**d**) were assessed following the procedures outlined in the Materials and Methods section (n = 3, respectively). Top photo of panel **c:** representative result image. Data are presented as mean percentages ± SEMs of 2% FBS control levels. * *p* < 0.05 vs. cells exposed to α-MSH alone and 2% FBS.

Initially, we investigated the effects of AESFEO at concentrations of 10 to 250 μg/mL on B16BL6 melanoma cell viability. AESFEO had no significant effect at 10 to 200 μg/mL, but at 250 μg/mL, it inhibited B16BL6 cell viability significantly (Figure 4a). Thus, AESFEO concentrations of 10 to 200 μg/mL were selected to test other effects of AESFEO. Next, we used WST assays to determine whether AESFEO affects B16BL6 cell proliferation. Treatment with 200 μg/mL AESFEO significantly suppressed 2% FBS-induced proliferation (from 177.67 ± 5.28 to 144.67 ± 8.65% versus untreated controls; Figure 4b).

To assess the effect of AESFEO on melanin synthesis in B16BL6 cells, we treated α-MSH (200 nM)-exposed B16BL6 cells in the presence of FBS (2%) with 10–200 μg/mL of AESFEO and then analyzed melanin contents. AESFEO at concentrations of 50 to 200 μg/mL significantly reduced melanin synthesis in α-MSH-exposed B16BL6 cells and had the greatest effect at 200 μg/mL (from 240.71 ± 0.00 to 108.52 ± 4.72% versus 2% FBS control; Figure 4c). We also analyzed the effect of AESFEO (10–200 μg/mL) on tyrosinase activity in α-MSH (200 nM)-treated B16BL6 cells. Tyrosinase activity level in these cells was significantly and concentration-dependently inhibited by AESFEO (50–200 μg/mL), and this effect was greatest at 200 μg/mL (from 289.96 ± 4.78 to 136.32 ± 14.39% of the 2% FBS control; Figure 4d).

Excessive melanin production can cause many skin problems [15], and thus, many researchers have sought means of suppressing melanin biosynthesis. Studies have shown that this can be achieved by regulating melanocyte survival, differentiation, or proliferation [44,45]. For example, Zhang et al. reported suppressing melanocyte proliferation inhibited melanogenesis [46]. On the other hand, Maeda et al. concluded that melanogenesis is correlated with tyrosinase activity and level [47], and Han and Hyun observed tyrosinase activity was positively associated with melanin production in melanocytes [29]. These reports suggest that the suppression of tyrosinase activity and melanocyte proliferation inhibit melanogenesis. In the current study, we found that AESFEO reduced serum-induced increases in the proliferation of B16BL6 cells and attenuated tyrosinase activity in α-MSH-stimulated B16BL6 cells. We also observed that AESFEO reduced melanin levels in B16BL6 cells exposed to α-MSH. Therefore, our findings imply that AESFEO has the potential to suppress melanin synthesis by melanocytes by inhibiting cell proliferation and tyrosinase activity.

### 3.5. Effect of AESFEO on Melanogenesis Regulation-Related Proteins in B16BL6 Cells

**Figure 5:**
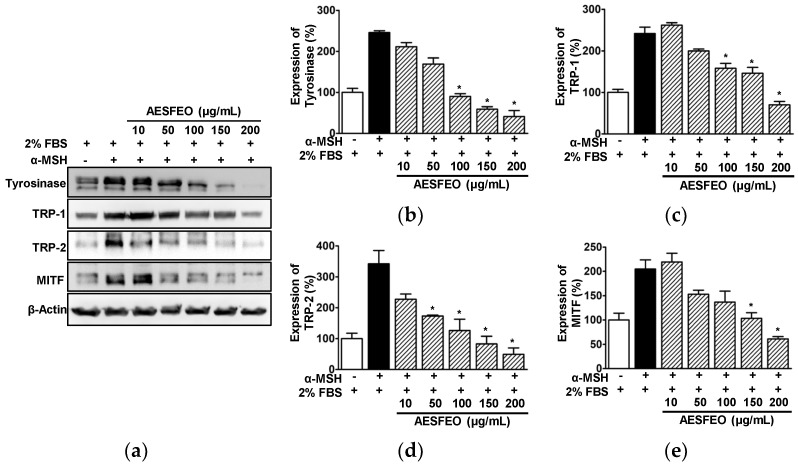
Effects of AESFEO on proteins related to melanogenesis in B16BL6 melanoma cells. (**a**) Representative images. B16BL6 cells were cultured in medium containing 2% FBS, with α-MSH (200 nM) either present or absent, and with and without *Aralia elata* (Miq.) Seem. flower absolute-type essential oil (AESFEO; 10–200 μg/mL) for 24 h. Cell lysates were Western blotted with each antibody as in Materials and Methods. (**b**–**e**) Graphs showing tyrosinase (n = 3; **b**), TRP-1 (n = 3; **c**), TRP-2 (n = 3; **d**), and MITF (n = 3; **e**) expression levels. Protein expression levels are presented as mean percentages ± SEMs of levels in 2% FBS treated controls. * *p* < 0.05 vs. cells exposed to α-MSH in the presence of 2% FBS. TRP-1 and -2, tyrosinase-related protein-1 and -2; MITF, microphthalmia-associated transcription factor.

To determine whether AESFEO-induced inhibitions of melanogenesis and tyrosinase activity are associated with melanogenesis regulation-related proteins, we examined the effect of AESFEO (10–200 μg/mL) on the expressions of melanogenesis regulatory enzymes (tyrosinase, TRP-1 and TRP-2) and on MITF (the upstream regulator of these enzymes) in B16BL6 cells exposed to α-MSH (200 nM) in the presence of FBS (2%) by Western blot. AESFEO (100–200 μg/mL) significantly reduced the expression of tyrosinase in α-MSH-stimulated B16BL6 cells and had greatest effect at 200 μg/mL (from 245.50 ± 4.64 to 41.24 ± 14.48% of the 2% FBS control; Figure 5a,b). AESFEO also significantly inhibited the α-MSH-induced expressions of TRP-1 and TRP-2 in B16BL6 cells at 100–200 μg/mL (Figure 5a,c) and at 50 to 200 μg/mL (Figure 5a,d), respectively. The effects of AESFEO on TRP-1 and TRP-2 expressions in α-MSH-exposed B16BL6 cells were greatest at 200 μg/mL (from 241.90 ± 15.13 to 70.04 ± 8.13% of the 2% FBS control [Figure 5a,c] and from 315.55 ± 29.37 to 53.25 ± 10.34% of the 2% FBS control [Figure 5a,d], respectively). In addition, AESFEO at 150 and 200 μg/mL significantly reduced MITF expression in α-MSH-exposed B16BL6 cells, and this effect was greatest at 200 μg/mL (from 204.66 ± 18.87 to 61.15 ± 4.80% of the 2% FBS control; Figure 5a,e).

Reportedly, skin whitening can be induced by inhibiting tyrosinase, TRP-1, and TRP-2, key enzymes in melanin biosynthesis pathway [48,49]. In particular, tyrosinase has been suggested as a potential target for inhibiting melanin biosynthesis because it acts as the rate-limiting enzyme in the first step of melanin biosynthesis [50,51]. Lee et al. found that tyrosinase inhibition or downregulation attenuated melanin production in α-MSH-induced B16BL6 melanoma cells [52], and Joo et al. observed that the downregulation of TRP-1 and TRP-2 suppressed melanogenesis in B16F10 cells exposed to α-MSH [53]. In the present study, treatment with AESFEO reduced the upregulation of tyrosinase, TRP-1, and TRP-2 proteins induced by α-MSH in B16BL6 cells. In addition, Kim et al. reported that the transcription factor MITF can regulate the expressions of tyrosinase, TRP-1, and TRP-2 [14], and Shin et al. confirmed this by showing that MITF downregulation inhibits the expressions of TRP-1, TRP-2, and tyrosinase and reduces melanin production by melanocytes [54]. In the present study, we found that AESFEO reduced MITF expression and melanin levels in α-MSH-stimulated B16BL6 cells, implying that AESFEO has anti-melanogenesis effects through MITF inhibition. Therefore, our findings suggest that AESFEO suppresses melanogenesis by downregulating the expressions of MITF, tyrosinase, TRP-1, and TRP-2 in melanocytes.

### 3.6. MAPK Phosphorylations in AESFEO-Treated B16BL6 Cells

**Figure 6:**
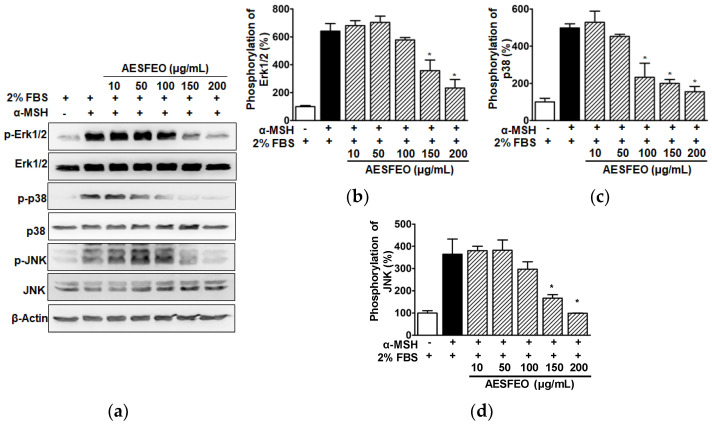
Effect of AESFEO on MAPK phosphorylation in B16BL6 cells. (**a**) Representative images. Cells were cultured in the presence of 2% FBS with and without *Aralia elata* (Miq.) Seem. flower absolute-type essential oil (AESFEO; 10–200 μg/mL) in the presence or absence of α-MSH (200 nM) for 5 min. Cell lysates were Western blotted with each antibody as described in Materials and Methods. (**b**–**d**) Graphs of phosphorylated Erk1/2 (**b**; n = 3), p38 MAPK (**c**; n = 3), and JNK levels (**d**; n = 3) resulted from panel (**a**). α-MSH represent a positive control. Phosphorylation levels of kinases are represented as percentages relative to those in 2% FBS-exposed controls. Results are expressed as means ± SEMs. * *p* < 0.05 vs. cells treated with α-MSH alone in the presence of 2% FBS. p-Erk1/2, phosphorylated Erk1/2; p-p38, phosphorylated p38 MAPK; p-JNK, phosphorylated JNK.

To determine whether AESFEO affects MAPKs known to be involved in melanogenesis, we analyzed the effect of AESFEO (10–200 μg/mL) on Erk1/2, p38 MAPK, and JNK phosphorylation in B16BL6 cells exposed to α-MSH (200 nM) in the presence of FBS (2%) by Western blot. Treatment of α-MSH-stimulated B16BL6 cells with AESFEO significantly attenuated Erk1/2 phosphorylation at 150 and 200 μg/mL (Figure 6a,b), p38 MAPK phosphorylation at 100–200 μg/mL (Figure 6a,c), and JNK phosphorylation at 150 and 200 μg/mL (Figure 6a,d). In all cases, 200 μg/mL AESFEO had maximal inhibitory effect.

Erk1/2, JNK, and p38 MAPK are important signal molecules that can control MITF expression and thus participate in the melanin production pathway in melanocytes [55,56], which indicates these MAPKs lie upstream of MITF in the melanin production pathway. Furthermore, it has been reported that increased Erk1/2 phosphorylation downregulates the expressions of MITF and melanogenic proteins and thus reduces melanogenesis in melanocytes [56,57]. However, increased Erk1/2 phosphorylation was also reported to promote melanin synthesis by downregulating MITF in melanocytes [58,59]. Therefore, the function of Erk1/2 activation in the melanogenic pathway remains controversial. On the other hand, the downregulations of p38 MAPK and JNK phosphorylation in melanocytes decreased melanogenesis by downregulating the expressions of MITF and melanogenic proteins [56]. In addition, p38 MAPK and JNK inhibitors decreased melanogenesis by reducing MITF and tyrosinase levels in B16F10 cells [55]. In the present study, AESFEO reduced Erk1/2, p38 MAPK, and JNK phosphorylation in B16BL6 cells exposed to α-MSH. As mentioned above, AESFEO also reduced melanin production, melanogenic enzyme levels, and MITF expression in α-MSH-exposed B16BL6 cells. Therefore, our results suggest that AESFEO might inhibit the activation of MAPKs in melanocytes and thus reduce melanogenic protein levels, including MITF, and inhibit melanogenesis.

### 3.7. Chemical Composition of AESFEO

**Figure 7:**
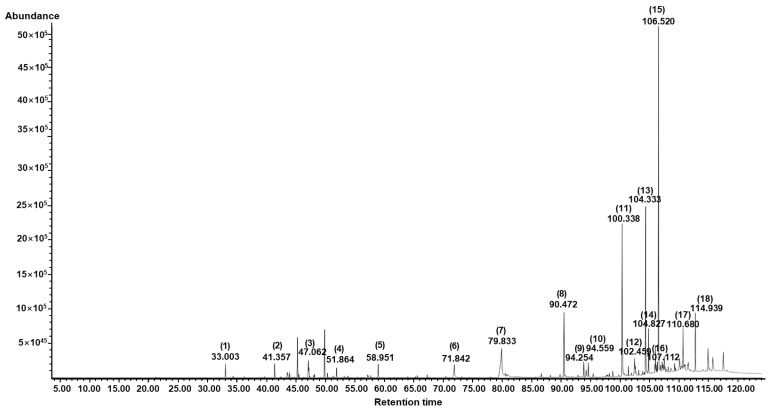
GC/MS total ion chromatogram of *Aralia elata* (Miq.) Seem. flower absolute-type essential oil. Peak numbers indicate compounds (bracketed), and corresponding retention times (below bracketed numbers) are shown in Table 1.

GC/MS analysis was conducted to identify the chemical composition of AESFEO, and 18 compounds were identified (Figure 7 and Table 1). The top four compounds based on GC peak areas were γ-tocopherol (26.93%), cyclotetracosane (14.31%), 1-docosene (14.00%), and linolenic acid (11.85%). Other compounds identified contained the antioxidant BKF (7.51%), δ-tocopherol (3.40%), α-amyrin (3.17%), dl-tetramisole (2.99%), hexadecanoic acid (2.87%), muscalure (2.28%), etc. (Table 1).

Of the compounds identified, hexadecanoic acid (also called palmitic acid) has been reported to induce keratinocyte proliferation [60,61], whereas α-amyrin stimulated HaCaT cells proliferation, and, thus, was suggested to have potential use for wound healing and skin regeneration [62]. DiPersio et al. concluded that keratinocyte migration and proliferation are essential for re-epithelization of the skin during wound healing [31], which suggests they may have been responsible for the keratinocyte proliferation effect of AESFEO. However, the effects of the other 16 identified components on wound-healing-related keratinocyte activities have not been reported. This topic needs to be further explored.

In addition, several of the 18 components of AESFEO have been reported to influence the melanogenesis-related responses of melanocytes associated with skin whitening. Farnesol and linolenic acid were reported to inhibit melanocyte proliferation [63,64,65], which suppresses melanogenesis in melanocytes [46]. β-Caryophyllene, hexadecanoic acid, linolenic acid, δ-tocopherol, and γ-tocopherol decreased melanogenesis-related responses (tyrosinase activity, tyrosinase expression, TRP-1, TRP-2, and MITF expressions, or melanin production) in melanocytes [66,67,68,69,70,71,72]. Accordingly, these compounds might contribute to the inhibitory effects of AESFEO on melanogenesis-related responses in melanocytes. However, further research is required to identify the compounds in AESFEO responsible for its inhibition of melanogenesis-related responses in melanocytes.

## 4. Conclusions

In the present study, we found that, in HaCaT cells, AESFEO induced proliferation and collagen type IV synthesis and enhanced the phosphorylation of MAPKs (Erk1/2, p38 MAPK, JNK) and Akt. The skin-wound-healing-related responses in HaCaT cells were maximal upon treatment with 100 or 250 μg/mL AESFEO. In addition, AESFEO decreased cell proliferation induced by serum and inhibited melanin production and tyrosinase activity stimulated by α-MSH in B16BL6 cells. AESFEO also reduced the α-MSH-induced expressions of MITF, tyrosinase, TRP-1, TRP-2, which are melanogenic proteins, and downregulated the α-MSH-stimulated phosphorylation of MAPKs (Erk1/2, p38 MAPK, and JNK) in these cells. The anti-melanogenesis-related responses in B16BL6 cells were maximal upon treatment with 200 μg/mL AESFEO. Additionally, AESFEO was found to contain 18 compounds through GC/MS. These findings indicate that AESFEO is likely to have positive effects on skin wound healing and whitening-associated activities of keratinocytes and melanocytes, respectively. This study suggests that AESFEO should be considered a potential natural material for developing agents that promote skin wound healing and/or whitening activities. However, this study has some limitations in the use of AESFEO in developing the agents. In this study, we did not definitively identify the key bioactive components responsible for the observed effects of AESFEO on keratinocytes and melanocytes. Additionally, while AESFEO exhibited promising results in vitro, these results were not validated through in vivo testing. Furthermore, cell–cell communications using primary keratinocytes and melanocytes were not elucidated. Therefore, addressing these limitations will be necessary in future studies to enhance the practical applications of AESFEO.

## Figures and Tables

**Table 1:** Components of the absolute-type essential oil from *Aralia elata* (Miq.) Seem. flowers.

No	Component Name	RT ^1^	RI ^2^		Area (%)	CAS No.
			Observed	Literature		
**1**	l-Bornyl acetate	33.00	1280	1280	1.37	5655-61-8
**2**	Caryophyllene	41.36	1410	1418	1.42	87-44-5
**3**	α-Bisabolene	47.06	1503	1547	1.78	25532-79-0
**4**	β-Bisabolene	51.86	1585	1509	1.01	495-61-4
**5**	Farnesol	58.95	1713	1713	1.60	4602-84-0
**6**	Hexadecanoic acid	71.84	1968	1984	2.87	57-10-3
**7**	Linolenic acid	79.83	2142	2199	11.85	463-40-1
**8**	Antioxidant BKF	90.47	2394	2398	7.51	119-47-1
**9**	1-Octadecene	94.25	2491	1800	0.98	112-88-9
**10**	Pentacosane	94.60	2500	2500	1.48	629-99-2
**11**	Cyclotetracosane	100.34	2697	2445	14.31	297-03-0
**12**	Muscalure	102.46	2798	2272	2.28	27519-02-4
**13**	1-Docosene	104.34	2902	2196	14.00	1599-67-3
**14**	δ-Tocopherol	104.83	2932	2951	3.40	119-13-1
**15**	γ-Tocopherol	106.52	3040	3055	26.93	7616-22-0
**16**	9-(β-Chlorostyryl)-10-(phenylethynyl)anthracene	107.11	3080	-	1.05	80364-59-6
**17**	α-Amyrin	110.68	3330	3376	3.17	638-95-9
**18**	dl-Tetramisole	114.94	3556	-	2.99	5036-02-2
Total Identified (%)					100.00	

^1^ RT: Retention time, ^2^ RT: Retention indices were determined using a DB-5MS capillary column.

## Data Availability

All data are provided in the manuscript.
